# Changes in haematological and serum biochemical parameter concentrations from the day of calving to ketosis onset in Holstein dairy cows during the postpartum period

**DOI:** 10.1186/s13620-025-00293-4

**Published:** 2025-03-19

**Authors:** Seungmin Ha, Seogjin Kang, Mooyoung Jung, Sang Bum Kim, Seongsoo Hwang, Jihwan Lee, Donghyeon Kim, Ki Choon Choi, Jinho Park

**Affiliations:** 1 Rural Development Administration, National Institute of Animal Science, Cheonan, 31000 Republic of Korea; 2 College of Veterinary Medicine, Jeonbuk National University, Iksan, 54596 Republic of Korea

**Keywords:** Dairy cows, Metabolic adaptation, Postpartum period, Haematological parameters, Serum biochemical parameters

## Abstract

**Background:**

Dairy cows commonly experience a negative energy balance (NEB) during early lactation as energy demands for maintenance and milk production exceed intake. Although most cows metabolically adapt to NEB and avoid ketosis, those that fail to adapt develop ketosis, which disrupts metabolism and reduces productivity. Haematological and serum biochemical parameters are crucial for understanding these metabolic disruptions. However, limited research has examined how these parameters change from calving to ketosis onset. This study aimed to investigate these changes, identify parameters associated with ketosis classification, and evaluate their implications for dairy cow health. Blood samples were collected from the jugular vein of Holstein cows and β-hydroxybutyrate (BHBA) was tested once every three days during the postpartum period (8 times in 21 days).

**Results:**

Cows were categorised into three groups based on their highest BHBA concentration: non-ketosis (NK; BHBA < 1.2 mmol/L; n = 75), subclinical ketosis (SCK; BHBA ≥ 1.2 mmol/L and < 3.0 mmol/L; n = 46), and clinical ketosis (CK; BHBA ≥ 3.0 mmol/L; n = 35). The NK group had the highest red blood cell and monocyte counts, red cell distribution width, and alanine transaminase (ALT) concentrations. However, this group had the lowest mean corpuscular volume, mean corpuscular haemoglobin, non-esterified fatty acid (NEFA), and total bilirubin concentrations on the day of calving and at ketosis onset, followed by the SCK and CK groups (*p* < 0.05). In the NK group, counts of neutrophils, monocytes, and eosinophils, along with NEFA and lactate dehydrogenase (LDH) concentrations, decreased from the day of calving to ketosis onset. Conversely, ALT, aspartate transaminase (AST), and magnesium concentrations increased in the SCK and CK groups (*p* < 0.05). The NK group had the most pronounced changes in glucose, triglyceride, and magnesium and the lowest BHBA, LDH, and AST concentrations, followed by the SCK and CK groups (*p* < 0.05).

**Conclusions:**

This study identified key haematological and serum biochemical changes associated with ketosis classification in dairy cows, highlighting metabolic adaptations in the NK group that mitigate ketosis risk and metabolic dysfunctions in the SCK and CK groups that develop ketosis. These findings provide practical markers for early detection and management of ketosis, supporting improved dairy cow health and productivity.

**Supplementary Information:**

The online version contains supplementary material available at 10.1186/s13620-025-00293-4.

## Background

A negative energy balance, which is generally associated with ketosis, develops in most dairy cows during early lactation, as their energy expenditure—such as for maintenance, milk production, and immune system activity—exceeds their energy intake [1–4]. Dairy cows experiencing negative energy balance typically undergo metabolic adaptations to this physiological state; however, failure to adapt adequately can result in ketosis [4]. This maladaptation is caused by abnormal changes in interactions between energy use sites (e.g., mammary gland, skeletal muscle, and other tissues), energy storage sites (adipose tissue), and the energy source processing site (liver) [4]. In dairy cows, the mammary tissues utilise 60–85% of all available glucose to synthesise milk and its components (i.e., lactose and adenosine triphosphate) [5, 6]. The development of ketosis is associated with increased yield of colostrum and milk during early lactation in the transition period [7, 8]. To meet the heightened energy requirements for lactation, intensive lipolysis occurs in the adipose tissue, leading to an increase in non-esterified fatty acids (NEFAs) in the blood [9]. However, impaired regulation of this metabolic process can cause excessive NEFA release, which is closely associated with the development of ketosis [10, 11]. NEFAs are processed via three pathways in the liver: complete oxidation through the tricarboxylic acid (TCA) cycle to produce energy, H_2_O, and CO_2_; incomplete oxidation through ketogenesis, which produces less energy and ketone bodies, such as β-hydroxybutyrate (BHBA), acetoacetate, acetone; and re-esterification resulting in triglycerides (TGs) production [12]. TGs are exported from the liver as very low-density lipoproteins or stored in the hepatocytes [13]. The TCA cycle provides energy and intermediates essential for gluconeogenesis. Furthermore, substrates such as propionate derived from ruminal fermentation, lactate, L-alanine, and glycerol contribute carbon skeletons required for gluconeogenesis [14]. However, excessive hepatic TG accumulation causes fatty liver, which in turn impairs liver functions, including gluconeogenesis [15]. Bovine ketosis develops when the hepatic capacity for complete oxidation is limited, and the export rate of very low-density lipoproteins from the liver is suppressed [14].

The increasing serum concentrations of NEFAs, along with their processing, lead to the development of oxidative stress [16, 17]. Oxidative stress is involved in the induction of insulin resistance, [18] and dairy cows generally experience increased oxidative stress associated with high serum concentrations of NEFA during the post-transition period [19]. Moreover, cows with ketosis have been reported to experience severe hepatic oxidative stress due to reduced hepatic activities of antioxidant enzymes, hepatic apoptosis, and insulin resistance [14, 20–23].

Based on the results of the haematological and serum biochemical analyses in our previous study, we indicated that dairy cows with severe oxidative stress and hepatic dysfunction on the day of calving could develop postpartum ketosis [24]. However, the study did not investigate haematological and serum biochemical parameters at ketosis onset. Understanding these parameters is essential for elucidating the pathophysiology of postpartum ketosis. To the best of our knowledge, no studies have simultaneously examined haematological and serum biochemical parameters both on the day of calving and at ketosis onset. Hence, we aimed to (1) investigate changes in the haematological and serum biochemical parameters from the calving date to ketosis onset, (2) identify the parameters associated with ketosis classification, and (3) determine the health implications of ketosis.

## Methods

### Experimental cows

The study was conducted on Holstein cows raised and calved on a farm at the National Institute of Animal Science, Cheonan, Republic of Korea. To ensure homogeneity, cows were included if they calved a Holstein calf at least once between January 2018 and March 2022, were milked twice daily during the first 21 days postpartum, and were free from any health conditions or interventions that could affect the study outcomes. Specifically, cows were excluded if they calved twins, experienced an abortion (dead calf more than 10 days before the due date) [25, 26], had a premature birth (live calf more than 10 days before the due date) [25, 26], were milked less than twice daily due to debilitated conditions, or had conditions such as mastitis, agalactia, theileriosis, milk fever, acidosis, or abomasal ulcer. Additionally, cows on medication or those receiving nutritional or microbial supplements (capsules or boluses) after calving were excluded. Ultimately, 156 Holstein cows met these criteria and were enrolled in the study. All cows had ad libitum access to the same total mixed ration consisting of concentrates, soybean meal, corn silage, alfalfa hay, timothy hay, enzymes, minerals, and vitamin additives.

### Blood sampling and case definitions

**Fig. 1:**
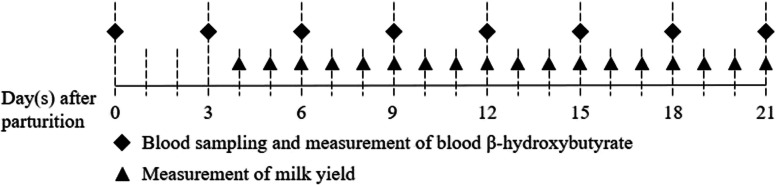
Study timeline: blood sampling and measurement of blood β-hydroxybutyrate concentration and milk yield. Blood sampling and measurement of blood β-hydroxybutyrate concentrations are performed eight times in the 21 days from the calving date (day 0). Milk yield is measured daily, starting 4 days after the calving date (day 4)

Blood samples were collected from the jugular vein of cows every three days during the postpartum period, totaling eight collections over the 21 days following calving (Fig. 1) [2]. The first sample was obtained between 6 and 23 h postpartum on the calving date. Subsequent samples were collected at three-day intervals (seven times), immediately after milking and coinciding with the commencement of feeding. Blood samples were collected in ethylenediaminetetraacetic acid (EDTA) tubes (BD Vacutainer K2 EDTA, BD, Franklin Lakes, NJ, USA) and serum-separating tubes (SSTs; BD Vacutainer SST II Advance, BD, Franklin Lakes, NJ, USA). BHBA concentration was determined immediately after blood sampling using an electronic handheld meter (FreeStyle Optium Neo, Abbott Diabetes Care Ltd., Witney, UK) and β-ketone test strips (FreeStyle Optium H β-Ketone, Abbott Diabetes Care Ltd., Witney, UK).

The cows were categorised into three groups according to the highest BHBA concentration in any of the eight postpartum samples as follows: the non-ketosis group [NK (BHBA < 1.2 mmol/L; n = 75)], subclinical ketosis [SCK (BHBA ≥ 1.2 mmol/L and < 3.0 mmol/L; n = 46)], and clinical ketosis [CK (BHBA ≥ 3.0 mmol/L; n = 35)] groups. The CK cows were treated as previously described [27]. Briefly, all cows were administered glycerin, and additional treatments included carnitine, vitamin B complex, vitamin E, and selenium, either individually or in combination.

### Blood analyses and data collection

Haematology and serum biochemistry analyses were performed at the National Institute of Animal Science laboratory located on the farm. The complete blood count (CBC) test was performed using a haematology analyser (Procyte Dx® haematology analyser, IDEXX Laboratories, Westbrook, MA, USA) with blood samples collected in EDTA tubes. The CBC was composed of erythrocyte, leukocyte, and platelet parameters. The erythrocyte parameters included red blood cell (RBC) count, haematocrit (Hct), haemoglobin (Hb), mean corpuscular volume (MCV), mean corpuscular haemoglobin (MCH), mean corpuscular haemoglobin concentration (MCHC), red cell distribution width (RDW), and reticulocyte count. The leukocyte parameters included white blood cell (WBC), neutrophil, lymphocyte, monocyte, eosinophil, and basophil counts. The platelet parameters included platelet count, mean platelet volume, platelet distribution width, and platelet crit.

The serum was separated by centrifuging SSTs at 3,000 rpm (2,600 g) for ten min, frozen, and stored at −70 ℃ pending analysis. Serum biochemical parameters were analysed in a single day using a biochemistry automatic analyser (Hitachi 7180, Hitachi Ltd., Tokyo, Japan). Glucose, NEFA, TG, total cholesterol (TC), total protein (TP), albumin, total bilirubin (TB), blood urea nitrogen (BUN), creatinine, alanine transaminase (ALT), aspartate transaminase (AST), alkaline phosphatase, γ-glutamyl transferase, lactate dehydrogenase (LDH), creatine kinase, calcium, magnesium, and inorganic phosphorus concentrations were measured using Hitachi 7180 after calibration and quality control assessments with commercial enzyme assay kits from Wako (Fujifilm Wako Pure Chemical Ltd., Osaka, Japan). Globulin concentration was calculated by subtracting albumin concentration from TP concentration. To determine the extent of the changes, we subtracted the values on the day of calving from those after ketosis onset.

Body condition scores (BCS) of dairy cows were assessed immediately following BHBA analysis of blood samples during the postpartum period, using the 5-point scale body condition scoring system [28]. The birth weight of neonatal calves was recorded immediately after parturition.

Daily milk yield was recorded from day four postpartum using an electronic milk meter (Alpro system, DeLaval, Tumba, Sweden). Milk production data from the first three days postpartum were not included in the analysis because, on this farm, colostrum was harvested in quantities sufficient only to feed the newborn calf rather than the full yield being collected (Fig. 1). The daily milk yield denotes the average of values for the previous three days.

The ketosis onset day was expressed relative to the day of calving, which was designated as day 0. For the NK group, it was defined as the day with the highest BHBA concentration. In the SCK group, it was defined as the first day when the BHBA concentration exceeded 1.2 mmol/L, and in the CK group, as the first day when the BHBA concentration exceeded 3.0 mmol/L.

### Statistical methods

Statistical analyses were performed using IBM SPSS Statistics for Windows, version 27.0 (IBM Corp., Armonk, NY, USA). The Shapiro–Wilk test and Levene test were used for normality analysis and equality of variances, respectively. For between-group comparisons, one-way analysis of variance (ANOVA) was applied to variables satisfying normality and equality of variances, with post-hoc comparisons conducted using the Bonferroni test. The Kruskal–Wallis test was used for variables that did not meet these assumptions, and post-hoc comparisons were performed using the Mann–Whitney U test with Bonferroni’s method, setting statistical significance at *p* < 0.017 (0.05/3) for three-group comparisons (NK-SCK, NK-CK, and SCK-CK). For within-group comparisons, paired *t*-tests were used for normally distributed variables, while the Wilcoxon signed-rank test was applied to non-normally distributed variables to assess changes from the calving date to the onset of ketosis. Data are expressed as mean ± standard deviation, with statistical significance set at *p* < 0.05 unless otherwise adjusted for multiple comparisons.

## Results

### Descriptive statistics for the Holstein cows by ketosis classification

The NK group had the lowest concentration of BHBA (0.51 ± 0.15 mmol/L) on the calving date, followed by the SCK (0.64 ± 0.23 mmol/L) and CK (0.74 ± 0.33 mmol/L) groups (*p* < 0.001; Fig. 1). All groups showed an increase in BHBA concentrations from the calving date to the day of ketosis onset; the increase in BHBA was lowest in the NK group (0.29 ± 0.18 mmol/L), followed by the SCK (0.81 ± 0.42 mmol/L) and CK (3.14 ± 0.80 mmol/L) groups (*p* < 0.001). At ketosis onset, the CK group had the highest BHBA concentration (3.88 ± 0.85 mmol/L), followed by the SCK (1.45 ± 0.31 mmol/L) and NK (0.80 ± 0.18 mmol/L) groups (*p* < 0.001). Ketosis onset occurred at 10.8 ± 5.3 days postpartum in the SCK group and at 11.0 ± 4.4 days postpartum in the CK group. At 11.0 ± 5.8 days postpartum, the NK group exhibited the highest BHBA concentration. The average age, birth weight of calves, BCS on the calving date, and milk yield from days 4 to 9 postpartum were highest in the CK group, followed by the SCK and NK groups, respectively (*p* < 0.05; Table 1). However, parity, the difference between the calving date and estimated due date, daily milk yield from days 10 to 21 postpartum, and the day of ketosis onset did not differ between the groups. BCS decreased from the calving date to the day of ketosis onset in all groups (by 0.08, 0.17, and 0.23 in the NK, SCK, and CK groups, respectively). The SCK group had the lowest BCS (2.90 ± 0.25) on the day of ketosis onset, followed by the NK (2.95 ± 0.25) and CK (3.10 ± 0.34) groups (*p* < 0.05).

**Table 1:** Characteristics of the Holstein cows included the study

Variable	NK	SCK	CK	*p*-value
Number	75	46	35	
Age at calving, years	4.20 ± 1.79^a^	4.73 ± 1.77^ab^	5.80 ± 2.56^b^	0.003
Parity	2.08 ± 1.18	2.39 ± 1.22	2.69 ± 1.59	0.111
Difference between calving date and due date, days	−1.43 ± 5.02	−1.80 ± 6.39	−2.00 ± 3.83	0.671
Calf Birth Weight, kg	41.0 ± 5.5 ^c^	42.0 ± 4.4^cd^	43.7 ± 5.3^d^	0.037
Body Condition Score				
Calving date	3.04 ± 0.27 ^a^	3.07 ± 0.30 ^a^	3.33 ± 0.34 ^b^	< 0.001
Onset day	2.95 ± 0.25 ^ab^	2.90 ± 0.25 ^a^	3.10 ± 0.34 ^b^	0.024
Daily milk yield (Days 4 – 21 postpartum), kg/day				
Day 4 – Day 6 postpartum	23.2 ± 7.1 ^c^	25.9 ± 6.8^cd^	28.2 ± 4.1 ^d^	0.005
Day 7 – Day 9 postpartum	27.5 ± 7.2 ^c^	29.8 ± 6.5^cd^	31.8 ± 7.1^d^	0.035
Day 10 – Day 12 postpartum	29.9 ± 7.9	32.1 ± 7.2	31.0 ± 7.3	0.366
Day 13 – Day 15 postpartum	31.6 ± 8.2	33.3 ± 6.4	32.6 ± 7.2	0.550
Day 16 – Day 18 postpartum	31.8 ± 8.8	34.7 ± 7.3	31.8 ± 6.8	0.179
Day 19 – Day 21 postpartum	33.0 ± 8.3	35.0 ± 7.9	33.2 ± 7.4	0.455
Onset day	11.0 ± 5.8	10.8 ± 5.3	11.0 ± 4.4	0.924
Number of cows				
Day of ketosis onset				
Day 0 (the day of calving)				
Day 3 postpartum	7	3	2	
Day 6 postpartum	21	13	6	
Day 9 postpartum	12	10	10	
Day 12 postpartum	12	6	6	
Day 15 postpartum	6	3	6	
Day 18 postpartum	7	9	5	
Day 21 postpartum	10	2		

The onset day signifies the day with the highest β-hydroxybutyrate concentration (in the NK group) or the day of ketosis onset (in the SCK and CK groups)

Data are expressed as the mean ± standard deviation values

*NK* non-ketosis group; *SCK* subclinical ketosis group; *CK* clinical ketosis group

^a,b^Different letters in the same row indicate significant differences (*p* < 0.017, Mann–Whitney U test with Bonferroni’s method)

^c,d^Different letters in the same row indicate significant differences (*p* < 0.05, Bonferroni’s test)

### Association between ketosis and haematological parameters on the calving date and the day of ketosis onset

**Fig. 2:**
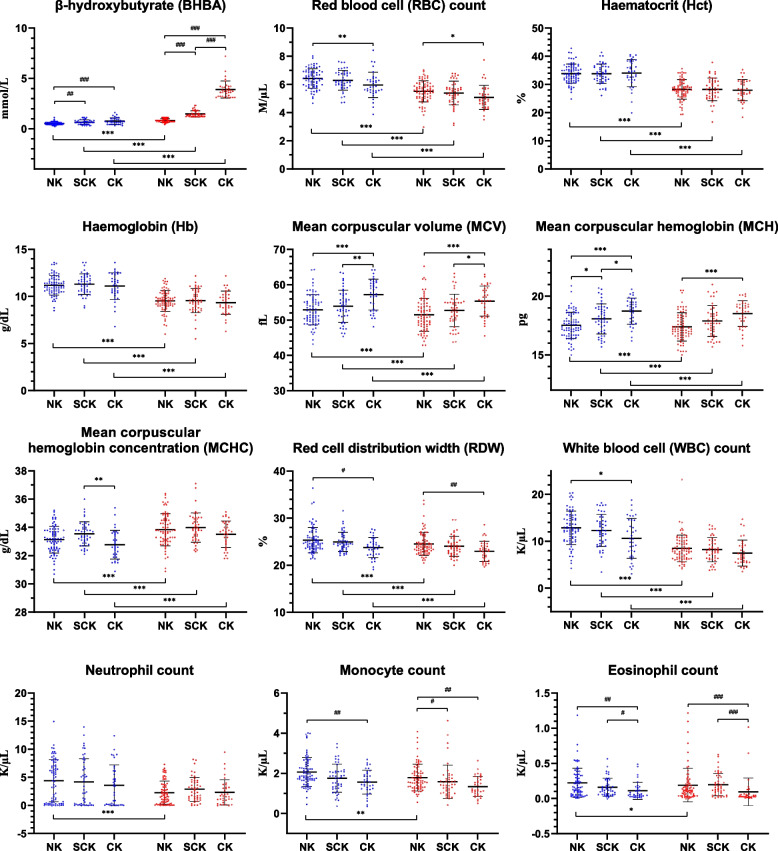
β-hydroxybutyrate concentration and haematological parameter values on the calving date and the day of ketosis onset according to ketosis classification. Results are expressed as mean ± standard deviation values. Error bars represent the standard deviation. The blue and red dots represent the values on the calving date and on the day of ketosis onset, respectively. Note: Onset signifies the day on which the β-hydroxybutyrate concentration is the highest (in the NK group) or the day of ketosis onset (in the SCK and CK groups). Abbreviations: NK, non-ketosis group; SCK, subclinical ketosis group; CK, clinical ketosis group. ^*^p < 0.05; ^**^p < 0.01; ^***^p < 0.001; ^#^*p* < 0.017; ^##^*p* < 0.003; ^###^*p* < 0.0003

Regarding haematological parameters, the NK group had the highest RBC (6.41 ± 0.69 M/µL), WBC (12.9 ± 3.6 K/µL), monocyte (2.08 ± 0.73 K/µL), and eosinophil (0.23 ± 0.21 K/µL) counts and RDW (25.3 ± 2.7%), and the lowest MCV (52.9 ± 4.3 fL) and MCH (17.5 ± 1.1 pg) concentrations, followed by the SCK (6.30 ± 0.71 M/µL, 12.2 ± 3.4 K/µL, 1.78 ± 0.71 K/µL, 0.17 ± 0.13 K/µL, 25.0 ± 2.1%, 53.9 ± 4.6 fL, and 18.1 ± 1.3 pg, respectively) and CK (5.95 ± 0.89 M/µL, 10.8 ± 4.3 K/µL, 1.58 ± 0.59 K/µL, 0.11 ± 0.12 K/µL, 23.8 ± 2.1%, 57.1 ± 4.3 fL, and 18.7 ± 1.1 pg, respectively) groups (*p* < 0.05; Fig. 2). All groups showed decreased RBC and WBC counts, Hct, Hb concentrations, MCV, MCH, and RDW, but increased MCHCs from the calving date to the onset of ketosis (*p* < 0.05). However, neutrophil, monocyte, and eosinophil counts decreased in the NK group from the calving date to the onset of ketosis (*p* < 0.05); conversely, these parameters did not differ in the SCK and CK groups. At the onset of ketosis, the NK group maintained the highest values of RBC (5.51 ± 0.75 M/µL) and monocyte (1.80 ± 0.67 K/µL) counts and RDW (24.5 ± 2.5%) and the lowest MCV (51.5 ± 4.7 fL) and MCH (17.4 ± 1.2 pg) concentrations, followed by the SCK (5.38 ± 0.86 M/µL, 1.57 ± 0.83 K/µL, 24.1 ± 2.1%, 52.7 ± 4.7 fL, and 17.9 ± 1.3 pg, respectively) and CK (5.08 ± 0.84 M/µL, 1.39 ± 0.53 K/µL, 23.0 ± 2.1%, 55.4 ± 4.2 fL, and 18.5 ± 1.1 pg, respectively) groups (*p* < 0.05). Meanwhile, none of the groups showed differences in Hct and Hb concentrations on the calving date or the day of ketosis onset.

### Association between ketosis and serum biochemical parameters on the calving date and the day of ketosis onset

**Fig. 3:**
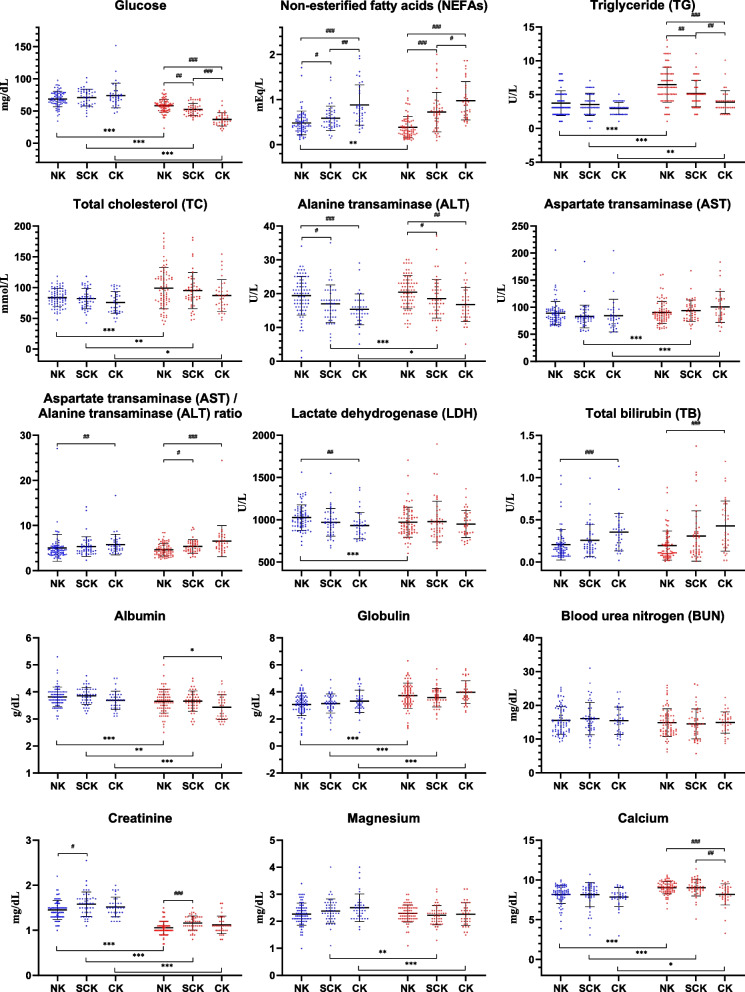
Serum biochemical parameter concentrations on the calving date and the day of ketosis onset according to ketosis classification. Results are expressed as mean ± standard deviation values. Error bars represent the standard deviation. The blue and red dots represent the values on the calving date and on the day of ketosis onset, respectively. Note: Onset signifies the day on which the β-hydroxybutyrate concentration is the highest (in the NK group) or the day of ketosis onset (in the SCK and CK groups). Abbreviations: NK, non-ketosis group; SCK, subclinical ketosis group; CK, clinical ketosis group. ^*^p < 0.05; ^**^p < 0.01; ^***^p < 0.001; ^#^*p* < 0.017; ^##^*p* < 0.003; ^###^*p* < 0.0003

**Fig. 4:**
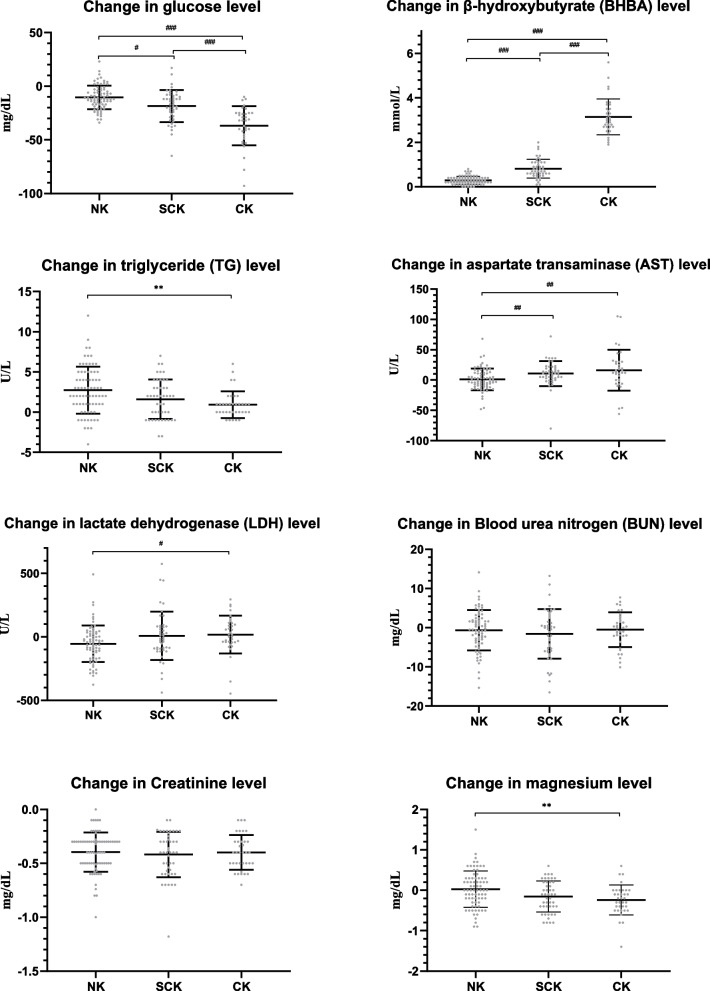
Changing extents of blood parameters according to ketosis classification. Results are expressed as mean ± standard deviation values. Error bars represent the standard deviation. Note: Onset signifies the day on which the β-hydroxybutyrate concentration is the highest (in the NK group) or the day of ketosis onset (in the SCK and CK groups). Abbreviations: NK, non-ketosis group; SCK, subclinical ketosis group; CK, clinical ketosis group. ^**^p < 0.01; ^#^*p* < 0.017; ^##^*p* < 0.003; ^###^*p* < 0.0003

On the calving date, the glucose concentration appeared numerically lower in the NK group compared to the SCK and CK groups; however, these differences were not statistically significant (*p* > 0.05; Fig. 3). The decrease in the glucose concentration from the calving date to the day of ketosis onset was the smallest in the NK group (10.5 ± 11.0 mg/dL), followed by the SCK (18.4 ± 14.9 mg/dL) and CK (36.9 ± 18.2 mg/dL) groups (*p* < 0.001; Fig. 4). On the day of ketosis onset, the NK group showed the highest concentration of glucose, followed by the SCK and CK groups (*p* < 0.001).

Regarding the lipid-related parameters, the NEFA concentration on the calving date was the lowest in the NK group, followed by the SCK and CK groups (*p* < 0.001; Fig. 3). The NEFA concentrations in the NK group decreased from the calving date to the onset of ketosis (*p* < 0.01), whereas those in the SCK and CK groups did not differ. The concentrations of TG and TC increased in all the groups from the calving date to the onset of ketosis (*p* < 0.05); however, the TG concentration increased the most in the NK group (2.73 ± 2.92 U/L), followed by those in the SCK (1.61 ± 2.44 U/L) and CK (0.94 ± 1.66 U/L) groups (Figs. 3 and 4). At the onset of ketosis, the CK group showed the highest NEFA and lowest TG concentrations, followed by the SCK and NK groups (*p* < 0.001).

Regarding the parameters related to enzymes and proteins, the NK group had the highest concentrations of ALT and LDH and the lowest TB concentration and AST/ALT ratio on the calving date, followed by the SCK and CK groups (*p* < 0.01; Fig. 3). Although AST did not differ between the groups on the calving date and ketosis onset, ALT and AST concentrations increased in the SCK and CK groups from the calving date to ketosis onset (*p* < 0.05), whereas those in the NK group did not differ (Figs. 3 and 4). In contrast to the results of ALT and AST, the NK group showed decreased LDH concentrations from the calving date to the onset of ketosis, whereas the SCK and CK groups showed no changes in LDH concentrations. The NK group maintained the highest ALT concentration and lowest TB concentration and AST/ALT ratio on the day of ketosis onset, followed by the SCK and CK groups (*p* < 0.01). The changes in BUN and creatinine concentrations between the calving date and ketosis onset did not differ between the groups (Fig. 4).

From the calving date to the onset of ketosis, the concentration of magnesium decreased in the SCK (−0.15 ± 0.39 mg/dL) and CK (−0.24 ± 0.37 mg/dL) groups (*p* < 0.01); the change in magnesium concentration was greatest in the CK group, followed by the SCK and NK groups (*p* = 0.004; Figs. 3 and 4). Calcium concentrations did not differ between the groups on the calving date. However, the concentrations increased from the calving date to the onset of ketosis. Calcium concentrations did not differ between the NK and SCK groups at the onset of ketosis, which were higher than the CK group (*p* < 0.002).

## Discussion

In this study, we investigated the associations between ketosis classification and blood parameters on the calving date and on the day of ketosis onset by categorising cows into the NK, SCK, and CK groups. On the calving date, ketosis classification was associated with RBC count, MCV, MCH, RDW, WBC count, monocyte count, eosinophil count, as well as the concentrations of BHBA, NEFA, ALT, LDH, TB, and AST-to-ALT ratio. Neutrophil, monocyte, and eosinophil counts, as well as NEFA and LDH concentrations, decreased in the NK group from the calving date to the day of ketosis onset, whereas ALT and AST concentrations increased and magnesium concentrations decreased in the SCK and CK groups. The changes in RBC count and Hct, Hb, BUN, and creatinine concentrations did not differ between the groups from the calving date to the onset of ketosis, whereas changes in BHBA, glucose, TG, AST, LDH, and magnesium concentrations were associated with ketosis classification. Once ketosis set in, its classification was associated with the RBC, monocyte, and eosinophil counts, MCV, MCH, RDW, concentrations of glucose, NEFA, TG, ALT, and TB, and AST-to-ALT ratio.

Polycythemia (elevated RBC count and Hct and Hb concentrations) and increased concentrations of BUN and creatinine indicate dehydration [29], and cows with ketosis are known to show signs of dehydration [30–32]. In this study, the Hct, Hb, and BUN concentrations were similar between the groups on the calving date and the onset of ketosis. Additionally, the changes in the extent of RBC count, Hct, Hb, BUN, and creatinine concentrations from the calving date to the onset of ketosis were similar. Based on the findings of this study, these results suggest that the dehydration observed as a clinical sign in previous studies is not associated with the progression of ketosis. Alternatively, dehydration might be a secondary consequence of advanced ketosis, occurring after its onset rather than contributing to its development.

Neutrophils, macrophages differentiated from monocytes, and eosinophils are involved in innate immunity [33]. These cells migrate from the bloodstream into tissues as part of the body’s immune response to infection and tissue damage. The innate immune system, including neutrophils and macrophages, responds to pathogens that cause mastitis and metritis, which frequently occur during the transition period [34–36]. Ketosis increases the risk of mastitis and metritis postpartum [37, 38]. Eosinophils, known to be involved in anti-parasitic responses and allergic reactions, dominate in bovine adipose tissue [39] and contribute to maintaining adipose tissue homeostasis [40]. In this study, cows with other diseases were excluded. In the NK group, neutrophil, monocyte, and eosinophil counts decreased from the calving date to ketosis onset, whereas no significant changes were observed in the SCK and CK groups. These differences in leukocyte changes may suggest a link between ketosis, susceptibility to other diseases, and adipose tissue homeostasis.

Ketosis is associated with insulin resistance, also known as impaired insulin sensitivity, in dairy cows [41]. Animals with insulin resistance fail to respond effectively to insulin and, consequently, exhibit elevated blood glucose concentrations [42]. Insulin resistance might explain the higher glucose concentrations observed on the calving date in cows with ketosis compared to those without ketosis. In cows, glucose obtained via a continuous and high rate of gluconeogenesis in the liver is utilised in the mammary glands to produce milk [43]. In this study, the CK group produced the most milk from days 4 to 9 postpartum, followed by the SCK and NK groups, which might be associated with a considerable decrease in glucose concentrations from the calving date to the onset of ketosis.

Insulin resistance also results in increased NEFA concentrations via tissue lipolysis [44]. The finding that the CK group had the highest NEFA concentrations on the calving date, followed by the SCK and NK groups, suggests that cows with ketosis might have experienced insulin resistance prior to its development. Under normal circumstances, the liver processes fatty acids by oxidation within the cell or secretion into the blood [45]. Although this mechanism is well-documented in general mammalian physiology, it is also applicable to cows. From the time of calving, cows with ketosis maintained NEFA concentrations higher than those in cows without ketosis, while NEFA concentrations in cows without ketosis decreased as lactation progressed. Considering that most dairy cows experience a negative energy balance [4] and that, in this study, all groups, regardless of their ketosis status, showed a decrease in BCS, these conditions indicate an increased mobilisation of NEFA from adipose tissue. The liver’s ability to process this NEFA load is critical to maintaining metabolic balance [2, 4]. The observation that some cows did not develop ketosis despite experiencing NEB and BCS loss suggests that ketone synthesis via incomplete oxidation of NEFA may be less prominent compared to complete oxidation and re-esterification in cows without ketosis. The result related to TG supports this suggestion, as cows with severe ketosis had very low serum TG concentrations.

In this study, ALT and AST concentrations increased in the ketosis (SCK and CK) groups, and the AST-to-ALT ratio was associated with ketosis at its onset. Although specific studies on AST and ALT in cows are lacking, findings from other species, including humans, provide valuable insights. AST and ALT are highly concentrated in hepatic cells and are involved in gluconeogenesis and the metabolism of amino acids and glucose [46]. Moreover, the relative AST-to-ALT ratio helps in the differential diagnosis of hepatic diseases [47]. Accelerated fatty acid oxidation results in the synthesis of enzymes involved in gluconeogenesis, including AST and ALT [48]. A high AST-to-ALT ratio tends to be associated with the utilisation of glucose as an energy source via glycolysis [49]. Furthermore, vitamin B_6_ deficiency decreases serum ALT concentrations and increases the AST-to-ALT ratio [47]. Based on these findings, the observed increases in ALT and AST concentrations, as well as the association of the AST-to-ALT ratio with ketosis, could indicate that ketosis in cows is linked to alterations in glucose metabolism or vitamin B_6_ deficiency. These conclusions, though derived from studies in other species, provide a plausible explanation for the observed biochemical changes in bovine ketosis.

Low serum magnesium concentration is associated with ketosis in cows [50]. Similarly, in humans, low serum magnesium concentration is linked to several liver diseases, and hepatic function can be improved by magnesium supplementation in some of these conditions [51]. Furthermore, in rats, magnesium decreases the effect of glucagon involved in hepatic glucose production, fatty acid oxidation, and lipolysis [52]. In this study, cows with ketosis exhibited a significant reduction in serum magnesium concentrations, while non-ketotic cows showed no notable changes. Notably, cows with more severe ketosis displayed an even greater decrease in serum magnesium concentrations. These findings suggest that magnesium may have a role in fatty acid oxidation as part of the metabolic adaptations to compensate for decreasing blood glucose concentrations, with the extent of magnesium utilisation appearing to increase in line with ketosis classification. However, further studies are needed to confirm the specific mechanisms involved in magnesium utilisation during ketosis.

We described the association between ketosis and blood parameters by examining their changes from the calving date to the onset of ketosis and proposed potential mechanisms involved in its development. However, a limitation of our study is that, although certain blood parameters have been identified as being associated with ketosis, their precise roles in the mechanisms underlying ketosis development remain unclear. Given the limited studies in dairy cows, we relied on findings from other species to support our interpretations, leading to speculative explanations. Therefore, further studies are needed to identify additional factors, including hormonal regulators, liver function, and lipid metabolism, and to clarify their specific roles in the mechanisms underlying ketosis development in Holstein cows.

## Conclusions

In this study, ketosis was associated with the values of RBC, monocyte, and eosinophil counts; MCV, MCH, and RDW; the concentrations of BHBA, NEFA, ALT, and TB both on the calving date and at the onset of ketosis during the postpartum period. Furthermore, neutrophil, monocyte, and eosinophil counts, as well as NEFA and LDH concentrations, did not decrease in ketotic cows from the calving date to the day of ketosis onset, unlike in healthy cows. In contrast, BHBA, ALT, and AST concentrations increased in ketotic cows, while glucose, TG, and magnesium concentrations decreased during this period. To the best of our knowledge, this study is the first to examine haematological and serum biochemical parameters on both the calving date and the onset of ketosis. Key findings include variations in immune cell dynamics and metabolic markers, which might highlight metabolic adaptations in healthy cows that mitigate ketosis risk and dysfunctions in ketotic cows that might exacerbate the condition. These findings might provide veterinarians with practical markers for early detection and intervention, while guiding researchers to explore mechanisms linking immune and metabolic dysfunctions to develop targeted therapies and nutritional strategies for improved dairy cow health and productivity.

## Supplementary Information

Supplementary Material 1.

## Data Availability

No datasets were generated or analysed during the current study.

## Abbreviations

ALT: Alanine transaminase
ANOVA: Analysis of variance
AST: Aspartate transaminase
BCS: Body condition score
BHBA: β-Hydroxybutyrate
BUN: Blood urea nitrogen
CBC: Complete blood count
CK: Clinical ketosis
Hb: Haemoglobin
Hct: Haematocrit
LDH: Lactate dehydrogenase
MCH: Mean corpuscular haemoglobin
MCHC: Mean corpuscular haemoglobin concentration
MCV: Mean corpuscular volume
NEFA: Non-esterified fatty acid
NK: Non-ketosis
RBC: Red blood cell
RDW: Red cell distribution width
SCK: Subclinical ketosis
SST: Serum-separating tubes
TB: Total bilirubin
TC: Total cholesterol
TCA: Tricarboxylic acid
TG: Triglyceride
TP: Total protein
WBC: White blood cell

## References

[1] Abuelo A, Mann S, Contreras GA. Metabolic Factors at the Crossroads of Periparturient Immunity and Inflammation. Vet Clini North Am Food AnimPract. 2023;39:203–18. 10.1016/j.cvfa.2023.02.012. 10.1016/j.cvfa.2023.02.012 37032303

[2] Drackley JK. Biology of dairy cows during the transition period: The final frontier? J Dairy Sci. 1999;82:2259–73. 10.3168/jds.S0022-0302(99)75474-3. 10575597 10.3168/jds.s0022-0302(99)75474-3

[3] Gross JJ, Bruckmaier R. Invited review: Metabolic challenges and adaptation during different functional stages of the mammary gland in dairy cows: Perspectives for sustainable milk production. J Dairy Sci. 2019;102:2828–43. 10.3168/jds.2018-15713. 30799117 10.3168/jds.2018-15713

[4] Herdt TH. Ruminant adaptation to negative energy balance: Influences on the etiology of ketosis and fatty liver. Vet Clin North Am Food Anim Pract. 2000;16:215–30. 10.1016/s0749-0720(15)30102-x. 11022337 10.1016/s0749-0720(15)30102-x

[5] Cant JP, Trout DR, Qiao F, Purdie NG. Milk synthetic response of the bovine mammary gland to an increase in the local concentration of arterial glucose. J Dairy Sci. 2002;85:494–503. 10.3168/jds.S0022-0302(02)74100-3. 11949851 10.3168/jds.S0022-0302(02)74100-3

[6] Annison EF, Bickerstaffe R, Linzell JL. Glucose and fatty acid metabolism in cows producing milk of low fat content. Journal Agric Sci. 1974;82:87–95. 10.1017/S0021859600050255.

[7] Vanholder T, Papen J, Bemers R, Vertenten G, Berge AC. Risk factors for subclinical and clinical ketosis and association with production parameters in dairy cows in the Netherlands. J Dairy Sci. 2015;98:880–8. 10.3168/jds.2014-8362. 25497823 10.3168/jds.2014-8362

[8] Ha S, Kang S, Jeong M, Han M, Lee J, Chung H, et al. Characteristics of Holstein cows predisposed to ketosis during the post-partum transition period. Vet Med Sci. 2023;9:307–14. 10.1002/vms3.1006. 36399368 10.1002/vms3.1006 PMC9857124

[9] Adewuyi AA, Gruys E, Van Eerdenburg FJ. Non esterified fatty acids (NEFA) in dairy cattle. A review Vet Q. 2005;27:117–26. 10.1080/01652176.2005.9695192. 16238111 10.1080/01652176.2005.9695192

[10] Xu Q, Li X, Ma L, Loor JJ, Coleman DN, Jia H, et al. Adipose tissue proteomic analysis in ketotic or healthy Holstein cows in early lactation. J Anim Sci. 2019;97:2837–49. 10.1093/jas/skz132. 31267132 10.1093/jas/skz132 PMC6606492

[11] Ning M, Zhao Y, Dai D, Yao C, Liu H, Fang L, et al. Gene co-expression network and differential expression analyses of subcutaneous white adipose tissue reveal novel insights into the pathological mechanisms underlying ketosis in dairy cows. J Dairy Sci. 2023;106:5018–28. 10.3168/jds.2022-22941. 37268588 10.3168/jds.2022-22941

[12] Roche JR, Kay JK, Friggens NC, Loor JJ, Berry DP. Assessing and managing body condition score for the prevention of metabolic disease in dairy cows. Vet Clin North Am Food Anim Prac. 2013;29:323–36. 10.1016/j.cvfa.2013.03.003. 10.1016/j.cvfa.2013.03.003 23809894

[13] Grummer RR. Etiology of lipid-related metabolic disorders in periparturient dairy cows. J Dairy Sci. 1993;76:3882–96. 10.3168/jds.S0022-0302(93)77729-2. 8132893 10.3168/jds.S0022-0302(93)77729-2

[14] White HM. The role of TCA cycle anaplerosis in ketosis and fatty liver in periparturient dairy cows. Animals (Basel). 2015;5:793–802. 10.3390/ani5030384. 26479386 10.3390/ani5030384 PMC4598706

[15] Zhang G, Ametaj BN. Ketosis an old story under a new approach. Dairy. 2020;1:42–60. 10.3390/dairy1010005.

[16] Miller JK, Brzezinska-Slebodzinska E, Madsen FC. Oxidative stress, antioxidants, and animal function. J Dairy Sci. 1993;76:2812–23. 10.3168/jds.S0022-0302(93)77620-1. 8227685 10.3168/jds.S0022-0302(93)77620-1

[17] Shi X, Li D, Deng Q, Li Y, Sun G, Yuan X, et al. NEFAs activate the oxidative stress-mediated NF-κB signaling pathway to induce inflammatory response in calf hepatocytes. J Steroid Biochem Mol Biol. 2015;145:103–12. 10.1016/j.jsbmb.2014.10.014. 25465477 10.1016/j.jsbmb.2014.10.014

[18] Bloch-Damti A, Bashan N. Proposed mechanisms for the induction of insulin resistance by oxidative stress. Antioxid Redox Signal. 2005;7:1553–67. 10.1089/ars.2005.7.1553. 16356119 10.1089/ars.2005.7.1553

[19] Sordillo LM, Raphael W. Significance of metabolic stress, lipid mobilization, and inflammation on transition cow disorders. Vet Clin North Am Food Anim Pract. 2013;29:267–78. 10.1016/j.cvfa.2013.03.002. 23809891 10.1016/j.cvfa.2013.03.002

[20] Du X, Chen L, Huang D, Peng Z, Zhao C, Zhang Y, et al. Elevated apoptosis in the liver of dairy cows with ketosis. Cell Physiol Biochem. 2017;43:568–78. 10.1159/000480529. 28934742 10.1159/000480529

[21] Bernabucci U, Ronchi B, Lacetera N, Nardone A. Influence of body condition score on relationships between metabolic status and oxidative stress in periparturient dairy cows. J Dairy Sci. 2005;88:2017–26. 10.3168/jds.S0022-0302(05)72878-2. 15905432 10.3168/jds.S0022-0302(05)72878-2

[22] Xu Chuang XC, Shu Shi SS, Xia Cheng XC, Wang Bo WB, Zhang HongYou ZH, Jun Bao JB. Investigation on the relationship of insulin resistance and ketosis in dairy cows. J Vet Sci Technol. 2014;5:162. 10.4172/2157-7579.1000162.

[23] Li Y, Ding HY, Wang XC, Feng SB, Li XB, Wang Z, et al. An association between the level of oxidative stress and the concentrations of NEFA and BHBA in the plasma of ketotic dairy cows. J Anim Physiol Anim Nutr (Berl). 2016;100:844–51. 10.1111/jpn.12454. 27079290 10.1111/jpn.12454

[24] Ha S, Kang S, Han M, Lee J, Chung H, Oh SI, et al. Predicting ketosis during the transition period in Holstein Friesian cows using hematological and serum biochemical parameters on the calving date. Sci Rep. 2022;12:853. 10.1038/s41598-022-04893-w. 35039562 10.1038/s41598-022-04893-w PMC8763895

[25] Murray RD. Abortion and perinatal mortality in cattle. In: Bovine Medicine. 3rd ed. John Wiley & Sons; 2015. p. 312–322.4

[26] Singh B, Kumar D. Incidence of abnormal termination of pregnancies in dairy cattle. Indian J Anim Reprod. 2007;28:59–66.

[27] Ha S, Kang S, Han M, Lee J, Chung H, Kim D, et al. Therapeutic Effects of Levocarnitine or Vitamin B Complex and E With Selenium on Glycerin-Treated Holstein Friesian Cows With Clinical Ketosis. Front Vet Sci. 2021;8: 773902. 10.3389/fvets.2021.773902. 34869746 10.3389/fvets.2021.773902 PMC8633306

[28] Edmonson A, Lean I, Weaver L, Farver T, Webster G. A body condition scoring chart for Holstein dairy cows. J Dairy Sci. 1989;72:68–78. 10.3168/jds.S0022-0302(89)79081-0.

[29] Latimer KS. Duncan and Prasse's veterinary laboratory medicine: clinical pathology. 5th ed. John Wiley & Sons; 2011.

[30] Garzón Audor AM, Oliver Espinosa OJ. Epidemiología de la cetosis en bovinos: una revisión. CES Med. Zootec. 2018;13:42–6129. 10.21615/cesmvz.13.1.4

[31] Tanzin AZ, Saifuddin A, Islam SA. Ketosis of an Early Lactating Crossbred Holstein-Friesian dairy cow: A Case Study. Bangladesh J Vet Anim Sci. 2020;8.

[32] McArt, J. A. A. Hyperketonemia in Cattle, 2024. https://www.merckvetmanual.com/metabolic-disorders/hyperketonemia-in-cattle/hyperketonemia-in-cattle

[33] Harvey JW. Veterinary hematology: a diagnostic guide and color atlas. 1st ed. Elsevier Health Sciences; 2011.

[34] Oviedo-Boyso J, Valdez-Alarcón JJ, Cajero-Juárez M, Ochoa-Zarzosa A, López-Meza JE, Bravo-Patiño A, et al. Innate immune response of bovine mammary gland to pathogenic bacteria responsible for mastitis. J Infect. 2007;54:399–409. 10.1016/j.jinf.2006.06.010. 16882453 10.1016/j.jinf.2006.06.010

[35] Pinedo PJ, Galvao KN, Seabury CM. Innate immune gene variation and differential susceptibility to uterine diseases in Holstein cows. Theriogenology. 2013;80:384–90. 10.1016/j.theriogenology.2013.04.027. 23768650 10.1016/j.theriogenology.2013.04.027

[36] Bradford BJ, Yuan K, Farney JK, Mamedova LK, Carpenter A. Invited review: Inflammation during the transition to lactation: New adventures with an old flame. J Dairy Sci. 2015;98:6631–50. 10.3168/jds.2015-9683. 26210279 10.3168/jds.2015-9683

[37] Suthar VS, Canelas-Raposo J, Deniz A, Heuwieser W. Prevalence of subclinical ketosis and relationships with postpartum diseases in European dairy cows. J Dairy Sci. 2013;96:2925–38. 10.3168/jds.2012-6035. 23497997 10.3168/jds.2012-6035

[38] Berge AC, Vertenten G. A field study to determine the prevalence, dairy herd management systems, and fresh cow clinical conditions associated with ketosis in western European dairy herds. J Dairy Sci. 2014;97:2145–54. 10.3168/jds.2013-7163. 24534510 10.3168/jds.2013-7163

[39] Bentley EG, Pugh G, Gledhill LR, Flynn RJ. An analysis of the immune compartment within bovine adipose tissue. Dev Comp Immunol. 2019;100: 103411. 10.1016/j.dci.2019.103411. 31202894 10.1016/j.dci.2019.103411

[40] Day KS, Rempel L, Rossi FMV, Theret M. Origins and functions of eosinophils in two non-mucosal tissues. Front Immunol. 2024;15:1368142. 10.3389/fimmu.2024.1368142. 38585275 10.3389/fimmu.2024.1368142 PMC10995313

[41] Hayirli A. The role of exogenous insulin in the complex of hepatic lipidosis and ketosis associated with insulin resistance phenomenon in postpartum dairy cattle. Vet Res Commun. 2006;30:749–74. 10.1007/s11259-006-3320-6. 17004039 10.1007/s11259-006-3320-6

[42] De Koster JD, Opsomer G. Insulin resistance in dairy cows. Vet Clin Food Anim Pract. 2013;29:299–322. 10.1016/j.cvfa.2013.04.002. 10.1016/j.cvfa.2013.04.002 23809893

[43] Wilke WL, Frandson RD, Fails AD. Anatomy and physiology of farm animals. 7th Ed. John Wiley & Sons; 2013.

[44] De Koster J, Nelli RK, Strieder-Barboza C, de Souza J, Lock AD, Contreras GA. The contribution of hormone sensitive lipase to adipose tissue lipolysis and its regulation by insulin in periparturient dairy cows. Sci Rep. 2018;8:13378. 10.1038/s41598-018-31582-4. 30190510 10.1038/s41598-018-31582-4 PMC6127149

[45] Alves-Bezerra M, Cohen DE. Triglyceride metabolism in the liver. Compr Physiol. 2017;8:1–8. 10.1002/cphy.c170012. 29357123 10.1002/cphy.c170012 PMC6376873

[46] Stockham SL, Scott MA. Fundamentals of veterinary clinical pathology. 2nd Ed. John Wiley & Sons; 2024.

[47] Giannini EG, Testa R, Savarino V. Liver enzyme alteration: a guide for clinicians. CMAJ. 2005;172:367–79. 10.1503/cmaj.1040752. 15684121 10.1503/cmaj.1040752 PMC545762

[48] Lam TK, Van de Werve G, Giacca A. Free fatty acids increase basal hepatic glucose production and induce hepatic insulin resistance at different sites. Am J Physiol Endocrinol Metab. 2003;284:E281–90. 10.1152/ajpendo.00332.2002. 12531742 10.1152/ajpendo.00332.2002

[49] Kobayashi A, Suzuki Y, Sugai S. Specificity of transaminase activities in the prediction of drug-induced hepatotoxicity. J Toxicol Sci. 2020;45:515–37. 10.2131/jts.45.515. 32879252 10.2131/jts.45.515

[50] Kaya A, Özkan C, Kozat S, Akgül Y, Özbek M. Evaluation of cobalt, copper, manganese, magnesium and phosphorus levels in cows with clinical ketosis. Pakistan Vet J. 2016;36:236–8.

[51] Liu M, Yang H, Mao Y. Magnesium and liver disease. Ann Transl Med. 2019;7:578. 10.21037/atm.2019.09.70 10.21037/atm.2019.09.70 PMC6861788 31807559

[52] Feng J, Wang H, Jing Z, Wang Y, Cheng Y, Wang W, et al. Role of magnesium in type 2 diabetes mellitus. Biol Trace Elem Res. 2020;196:74–85. 10.1007/s12011-019-01922-0. 31713111 10.1007/s12011-019-01922-0

