# Supplementary material for: Changes in haematological and serum biochemical parameter concentrations from the day of calving to ketosis onset in Holstein dairy cows during the postpartum period
Source: Ir Vet J. 2025 Mar 19;78:8. doi: 10.1186/s13620-025-00293-4 (PMC11921612; doi:10.1186/s13620-025-00293-4)
Supplement: Supplementary file 1 — Supplementary Material 1. [file 13620_2025_293_MOESM1_ESM.docx]

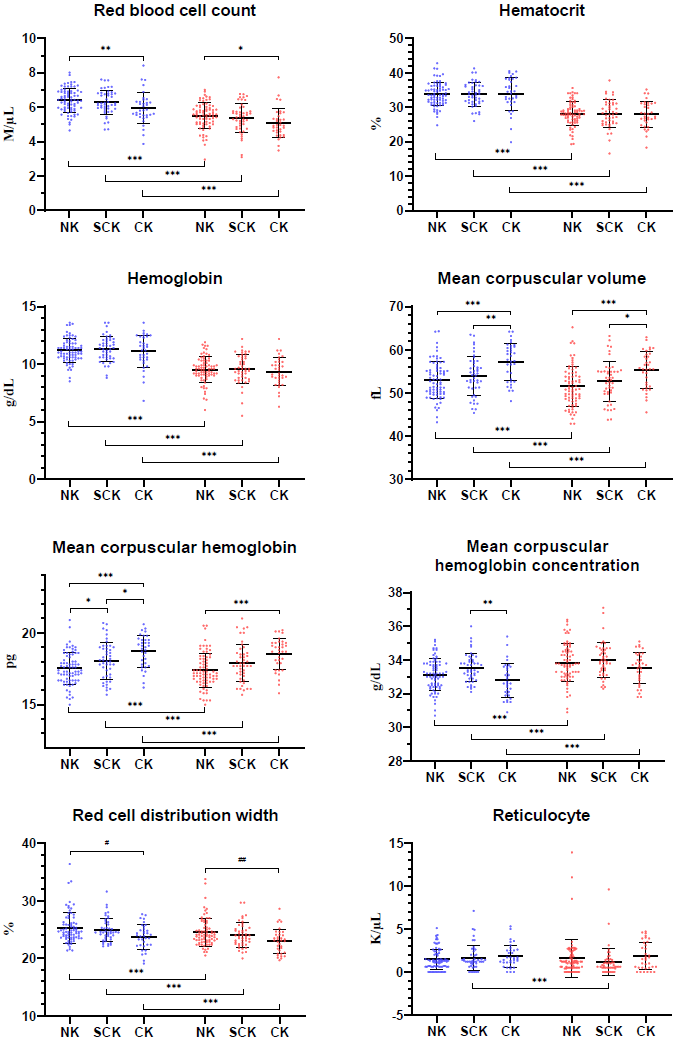


**Supplementary Fig. 1. Erythrocytes parameters according to the severity of ketosis.**

The results are expressed as mean ± standard deviation values. Error bars represent the standard deviation. The blue and red dots represent the values on the calving date and on the day of ketosis onset, respectively.

Note: Onset signifies the day on which the β-hydroxybutyrate level is the highest (in the NK group) or the day of ketosis onset (in the SCK and CK groups).

Abbreviations: NK, non-ketosis group; SCK, subclinical ketosis group; CK, clinical ketosis group

^*^*p*<0.05; ^**^*p*<0.01; ^***^*p*<0.001; ^#^*p*<0.017; ^##^*p*<0.003.


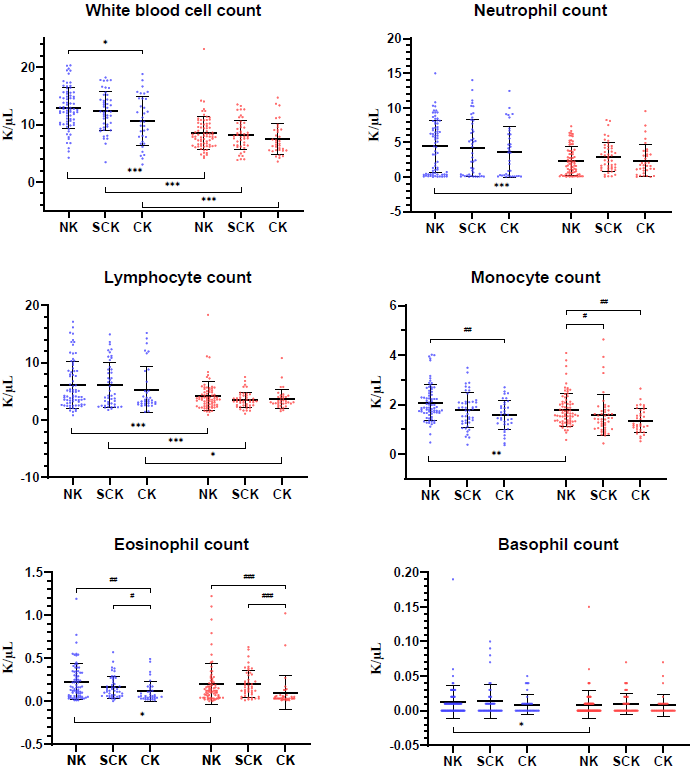


**Supplementary Fig. 2. Leukocyte parameters according to the severity of ketosis.**

The results are expressed as mean ± standard deviation values. Error bars represent the standard deviation. The blue and red dots represent the values on the calving date and on the day of ketosis onset, respectively.

Note: Onset signifies the day on which the β-hydroxybutyrate level is the highest (in the NK group) or the day of ketosis onset (in the SCK and CK groups).

Abbreviations: NK, non-ketosis group; SCK, subclinical ketosis group; CK, clinical ketosis group

^*^*p*<0.05; ^**^*p*<0.01; ^***^*p*<0.001; ^#^*p*<0.017; ^##^*p*<0.003; ^###^*p*<0.0003.


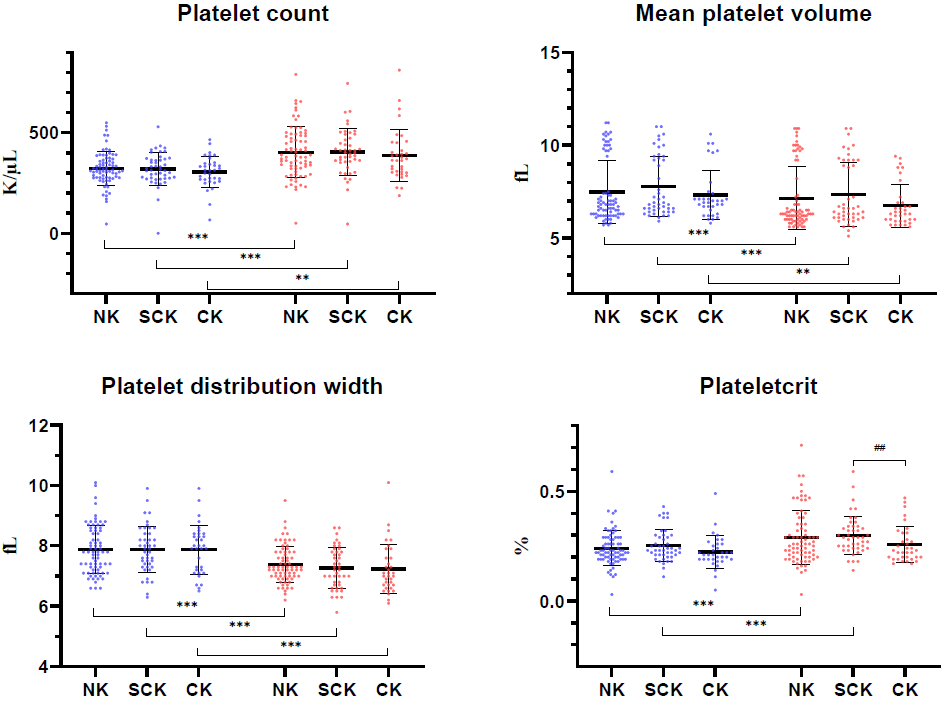


**Supplementary Fig. 3. Platelet parameters according to the severity of ketosis.**

The results are expressed as mean ± standard deviation values. Error bars represent the standard deviation. The blue and red dots represent the values on the calving date and on the day of ketosis onset, respectively.

Note: Onset signifies the day on which the β-hydroxybutyrate level is the highest (in the NK group) or the day of ketosis onset (in the SCK and CK groups).

Abbreviations: NK, non-ketosis group; SCK, subclinical ketosis group; CK, clinical ketosis group

^**^*p*<0.01; ^***^*p*<0.001; ^##^*p*<0.003.


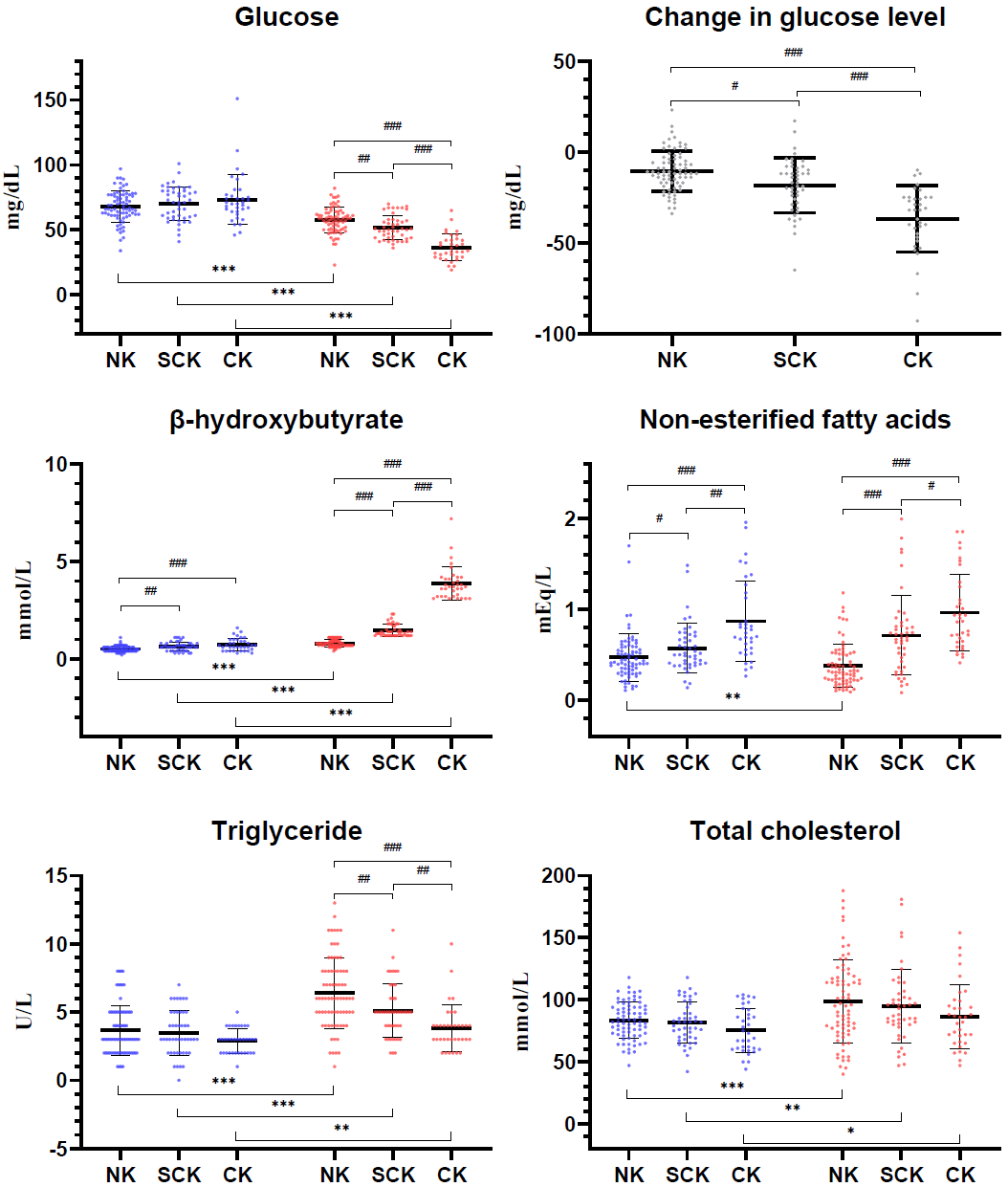


**Supplementary Fig. 4. Serum biochemical parameters related to glucose and lipids according to the severity of ketosis**

The results are expressed as mean ± standard deviation values. Error bars represent the standard deviation. The blue and red dots represent the values on the calving date and on the day of ketosis onset, respectively.

Note: Onset signifies the day on which the β-hydroxybutyrate level is the highest (in the NK group) or the day of ketosis onset (in the SCK and CK groups).

Abbreviations: NK, non-ketosis group; SCK, subclinical ketosis group; CK, clinical ketosis group

^*^*p*<0.05; ^**^*p*<0.01; ^***^*p*<0.001; ^#^*p*<0.017; ^##^*p*<0.003; ^###^*p*<0.0003.


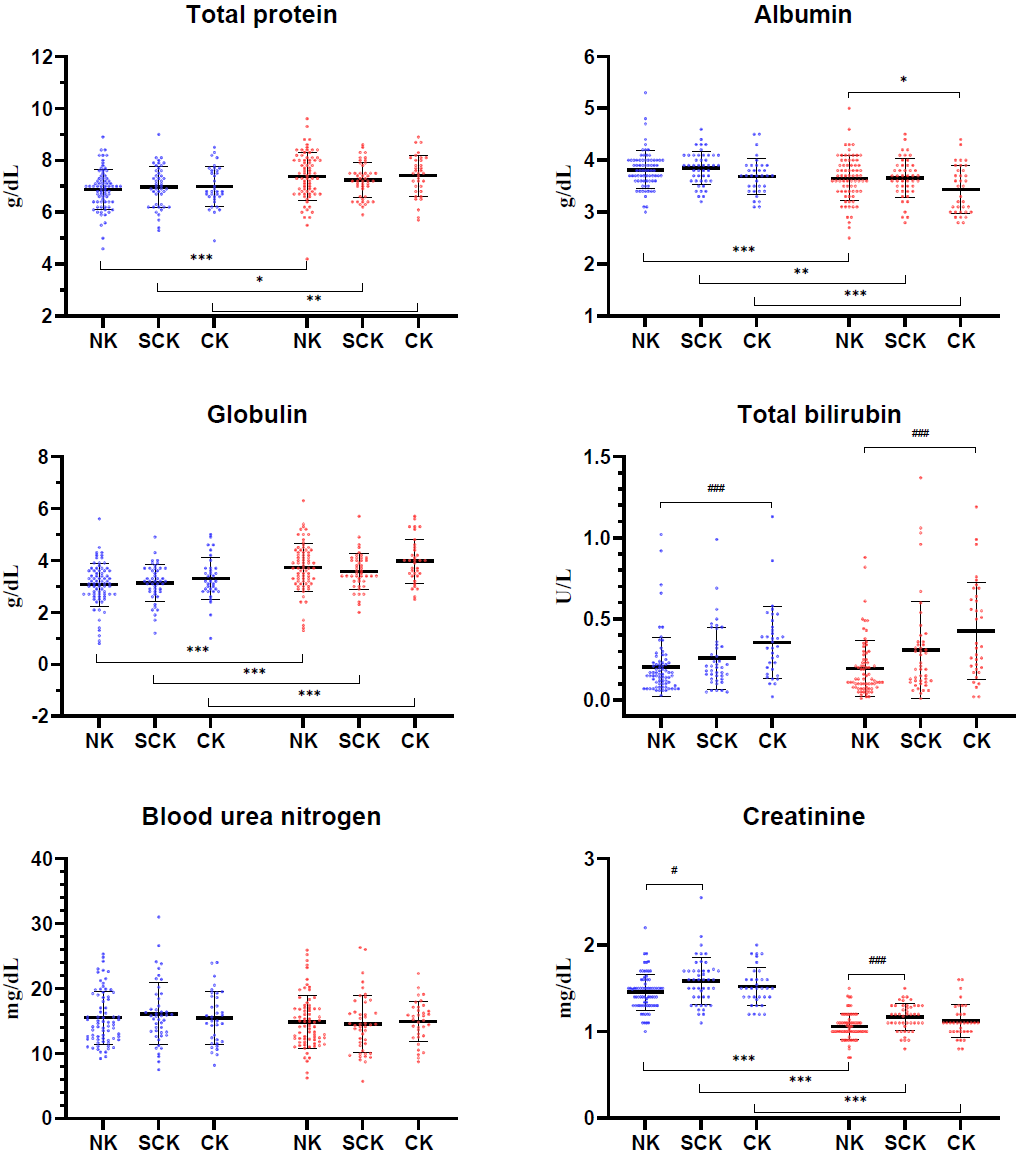


**Supplementary Fig. 5. Serum biochemical parameters related to proteins according to the severity of ketosis**

The results are expressed as mean ± standard deviation values. Error bars represent the standard deviation. The blue and red dots represent the values on the calving date and on the day of ketosis onset, respectively.

Note: Onset signifies the day on which the β-hydroxybutyrate level is the highest (in the NK group) or the day of ketosis onset (in the SCK and CK groups).

Abbreviations: NK, non-ketosis group; SCK, subclinical ketosis group; CK, clinical ketosis group

^*^*p*<0.05; ^**^*p*<0.01; ^***^*p*<0.001; ^#^*p*<0.017; ^###^*p*<0.0003.


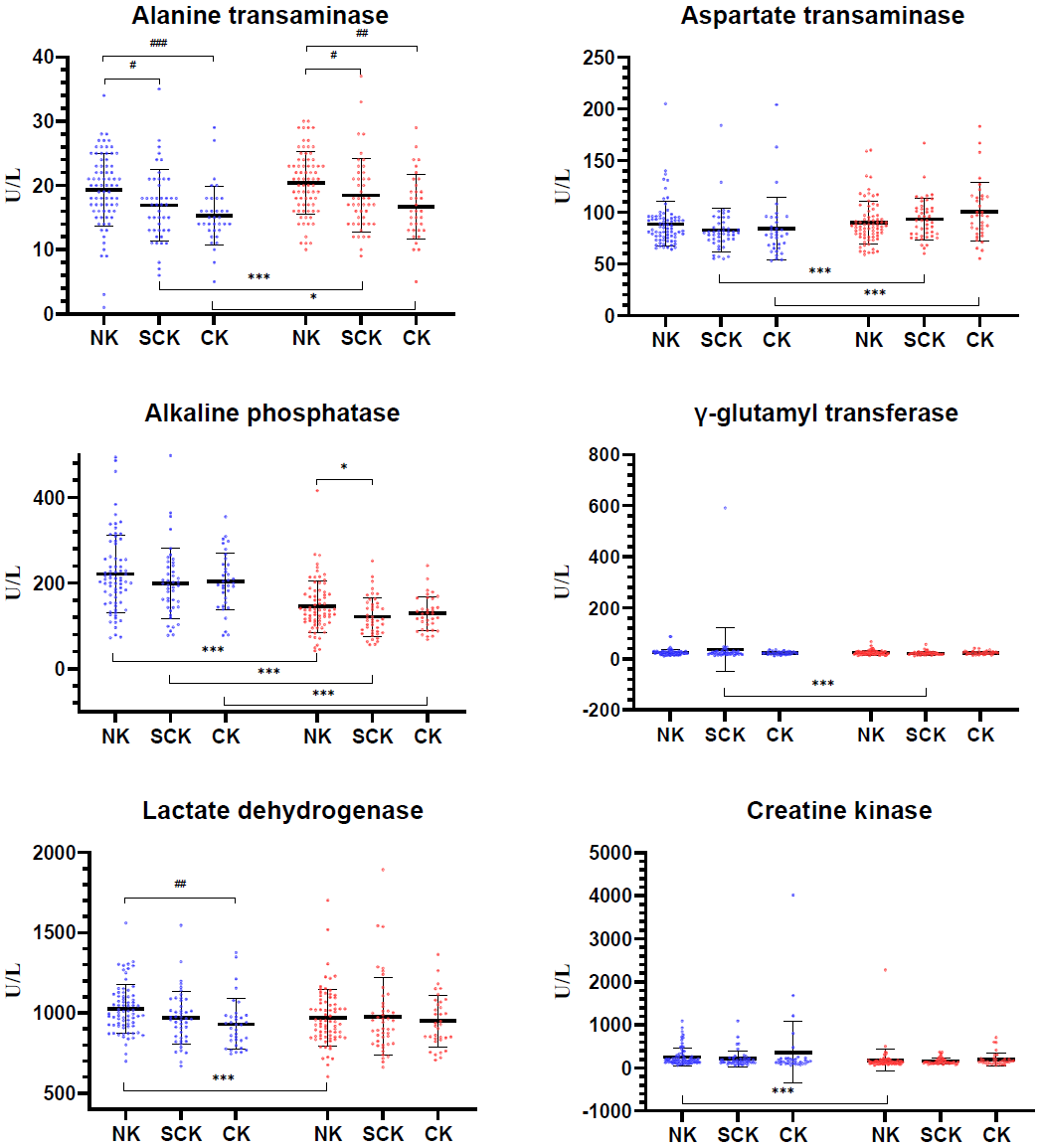


**Supplementary Fig. 6. Serum enzymatic biochemical parameters according to the severity of ketosis**

The results are expressed as mean ± standard deviation values. Error bars represent the standard deviation. The blue and red dots represent the values on the calving date and on the day of ketosis onset, respectively.

Note: Onset signifies the day on which the β-hydroxybutyrate level is the highest (in the NK group) or the day of ketosis onset (in the SCK and CK groups).

Abbreviations: NK, non-ketosis group; SCK, subclinical ketosis group; CK, clinical ketosis group

^***^*p*<0.001; ^#^*p*<0.017; ^##^*p*<0.003; ^###^*p*<0.0003.


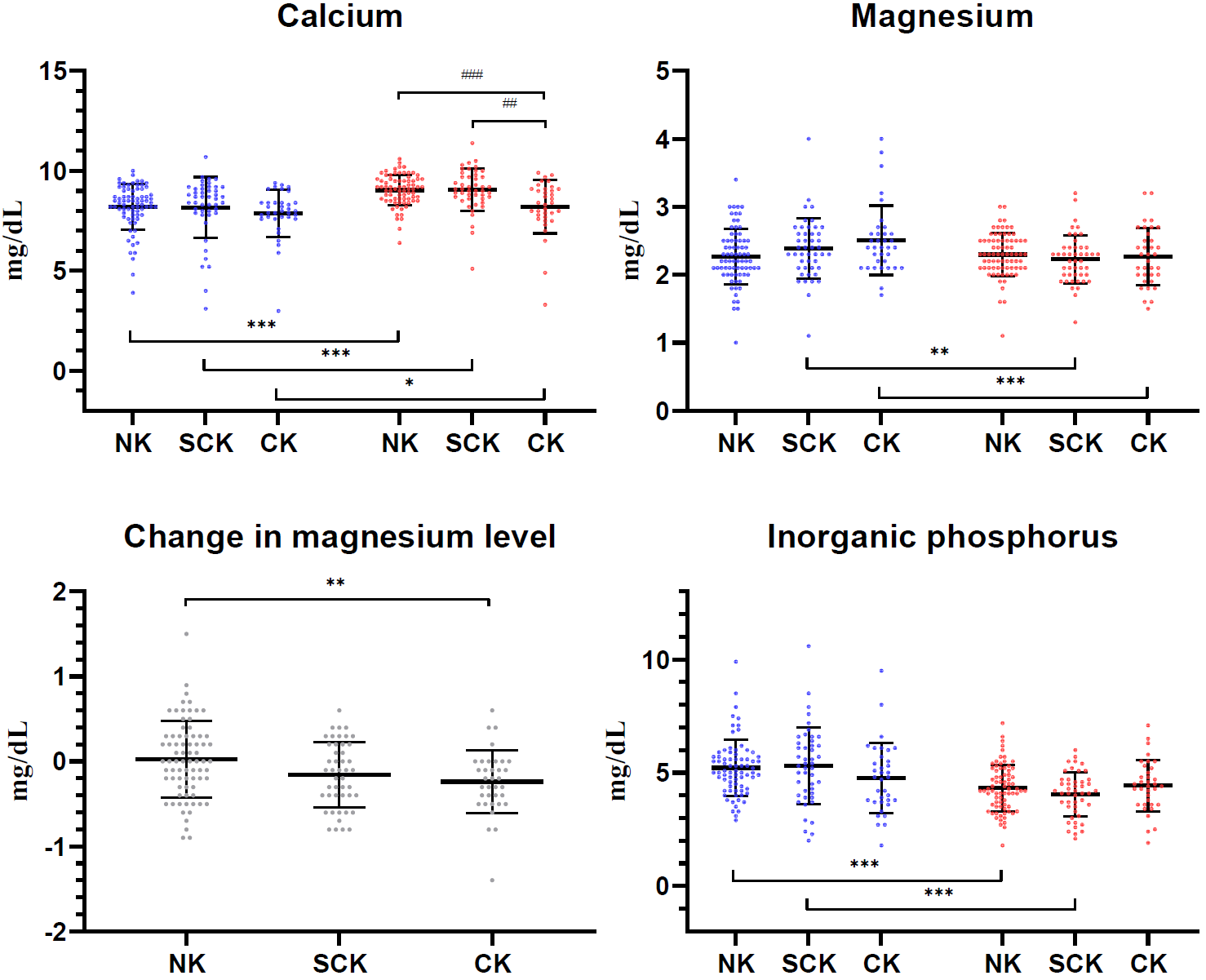


**Supplementary Fig. 7. Serum mineral parameters according to the severity of ketosis**

The results are expressed as mean ± standard deviation values. Error bars represent the standard deviation. The blue and red dots represent the values on the calving date and on the day of ketosis onset, respectively.

Note: Onset signifies the day on which the β-hydroxybutyrate level is the highest (in the NK group) or the day of ketosis onset (in the SCK and CK groups).

Abbreviations: NK, non-ketosis group; SCK, subclinical ketosis group; CK, clinical ketosis group

^*^*p*<0.05; ^**^*p*<0.01; ^***^*p*<0.001; ^##^*p*<0.003; ^###^*p*<0.0003.
